# Strength through diversity: how cancers thrive when clones cooperate

**DOI:** 10.1002/1878-0261.70160

**Published:** 2025-11-09

**Authors:** Marije C. Kuiken, Maartje Witsen, Emile E. Voest, Krijn K. Dijkstra

**Affiliations:** ^1^ Division of Molecular Oncology & Immunology The Netherlands Cancer Institute Amsterdam The Netherlands; ^2^ Oncode Institute The Netherlands Cancer Institute Amsterdam The Netherlands

**Keywords:** cancer progression, clonal cooperation, intratumoral heterogeneity, secreted factors, therapy resistance

## Abstract

Cancer is a highly heterogeneous disease, with many cancers containing multiple distinct subclones. While subclones are often seen as competitors (survival of the fittest), intratumor heterogeneity can also offer direct benefits to the tumor through cooperation between different clones. This has important clinical implications, as interdependent populations may present therapeutic vulnerabilities. Here, we review existing evidence for clonal cooperativity to address key questions and outline future developments based on six overarching principles: (a) secreted factors are important mediators of clonal cooperation; (b) (very) small subclones can significantly affect tumor behavior; (c) both genetic and nongenetic heterogeneity are substrates for cooperation; (d) nonmalignant cells from the tumor microenvironment can act as cooperating partners; (e) clonal cooperation occurs throughout different stages of cancer, from premalignancy to metastasis; and (f) clonal cooperation can promote therapy resistance by protecting otherwise sensitive populations. Together, these principles suggest clonal cooperation as an important mechanism in cancer. Lastly, we discuss how novel technological developments could address remaining gaps to open up new therapeutic strategies that exploit clonal cooperativity by targeting the tumor's weakest link.

## Graphical abstract

Intratumor heterogeneity can offer direct benefits to the tumor through cooperation between different clones. In this review, Kuiken et al. discuss existing evidence for clonal cooperativity to identify overarching principles, and highlight how novel technological developments could address remaining open questions. This has important clinical implications, as interdependent populations may present targetable therapeutic vulnerabilities.

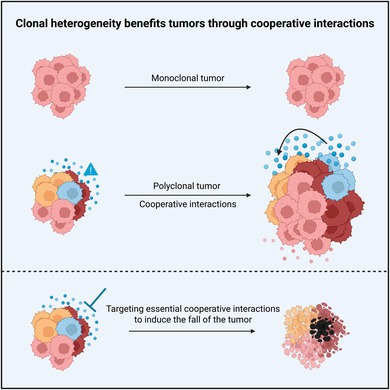

**Abbreviations**
CCL7: chemokine (C‐C) ligand 7
EGFR: epidermal growth factor receptor
EMT: epithelial‐to‐mesenchymal transition
FGF2: fibroblast growth factor 2
GF: growth factors
IFNγ: interferon gamma
IL‐6: interleukin‐6
ITH: intratumor heterogeneity
LIF: leukemia inhibitory factor
NK cells: natural killer cells
(non‐)NE: (non‐)neuroendocrine
OxR: oxaliplatin‐resistant
PD‐L1: programmed death ligand 1
PDX: patient‐derived xenografts
SCLC: small cell lung cancer
TGF‐α: transforming growth factor alpha
TGF‐β: transforming growth factor beta
TME: tumor microenvironment

## 1 Introduction

Intratumor heterogeneity (ITH) is pervasive throughout human cancers, with branched evolution—resulting in multiple coexisting subclones—the rule rather than the exception [1, 2]. High ITH is associated with worse prognosis and poor treatment outcome [3]. This raises the question of how the presence of multiple different subclones may benefit tumors. The field has traditionally focused on ITH as a substrate for selection, with different clones competing for survival, and ultimately the fittest subclones becoming dominant. However, tumors can also benefit more directly from ITH through the functional specialization of different clones that cooperate with each other [4]. Several studies have provided support for the concept of ‘clonal cooperativity’ in cancer [4, 5, 6, 7, 8, 9, 10, 11, 12]. While the notion of the cancer ecosystem has been widely embraced, most research focuses on interactions between cancer cells and nonmalignant cells (e.g., immune cells and fibroblasts) [13]. A better appreciation of how diverse subpopulations within the cancer cell compartment collectively shape the behavior of the tumor will be highly relevant to understand the dynamics of tumor progression and identify therapeutic vulnerabilities.

**Fig. 1:**
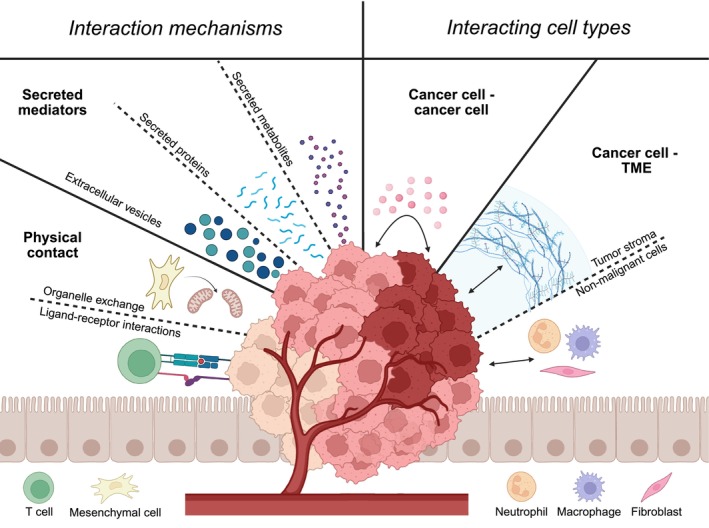
Overview of the different mechanisms and interacting cell types of cooperative interactions. Cooperative interactions can be mediated through physical contact (ligand–receptor interactions and organelle exchange) and through secreted factors (extracellular vesicles, secreted proteins, and secreted metabolites). Cooperative interactions can occur directly between different cancer subpopulations, or by modulating the stroma or non‐malignant cells in the TME. Figure created with BioRender.com.

Given the almost universal nature of ITH in cancer, it will be relevant to determine to what extent different clones engage in ‘ecological’ interactions. Similar to diverse species sharing an ecological habitat, interactions between clones can be negative or positive. Negative interactions include competition, where all parties negatively affect one another, and parasitism, where the interaction disadvantages one population while benefiting the other(s). Positive interactions include commensalism, where cooperation leaves one population unaffected while benefiting the other, and mutualism, where cooperation benefits all parties involved [14]. Clonal competition has been extensively reviewed elsewhere [15, 16], and this review will focus on cooperative interactions as an alternative paradigm to understand how clonal diversity determines the behavior of (human) cancers. We will describe the variety of mechanisms that can underlie clonal cooperativity, which includes both direct cooperation between different cancer subpopulations, as well as indirect effects through subclones that modulate nonmalignant cells and the microenvironment (Fig. 1).

## 2 A functional role for tumor heterogeneity

Several seminal studies in the early 2010s have laid the foundation for the concept of clonal cooperativity. Building on the notion that mutations in different genes cooperate to drive tumor growth, experiments in fruit fly models tested whether this could also occur if the different mutations were not present within the same cell, but rather in different cells. Wu et al. [12] indeed found that cells harboring mutations in the *scribbled* gene could non‐cell‐autonomously induce proliferation of neighboring *Ras* mutant clones, showing that distinct clones can cooperate to confer advantage to the tumor bulk. This was mediated by JNK signaling initiated by *scribbled* clones. Interestingly, JNK activation could also be induced by tissue damage, which indeed promoted the outgrowth of *Ras* mutant clones, showing that subclonal support in polyclonal tumors can mimic external stimuli. In a similar study on a *Ras* mutant background, also in fruit fly models, cells that carried mutations resulting in mitochondrial defects promoted the invasiveness and outgrowth of neighboring clones with intact mitochondrial function [17]. In both studies, the clones promoting proliferation of neighboring subclones were found at very low fractions in the eventual tumor, indicating their primary contribution to the tumor was non‐cell‐autonomous.

In these studies, genetically distinct clones were created by experimentally introducing mutations in *scribbled* and *Ras* genes. However, clonal cooperativity can also occur between naturally occurring heterogeneous populations. Calbo et al. [18] observed that murine small cell lung cancer (SCLC) frequently contain both neuroendocrine (NE) and nonendocrine subclones, that also differed at the genetic level, including a *Mycl1* amplification exclusively present in the NE clones. Through isolating these different populations, they showed that mesenchymal [non‐neuroendocrine (non‐NE)] cells endowed NE cells with metastatic properties when engrafted as a mixed population [18]. Neuroendocrine cells injected alone never metastasized, underscoring the relevance of subclonal cooperation in a mammalian model. In a different mouse model system, *Wnt1*‐driven breast cancers frequently contained *Hras*‐mutant (Wnt‐low) subclones. Despite the presence of a strong driver, these clones did not reach clonal dominance but depended on *Hras*‐wild‐type (Wnt‐high) subclones to propagate, illustrating that ITH can be essential for tumor growth when different clones benefit from each other [7].

These studies put the concept of clonal cooperativity on the map and challenge the paradigm that tumor evolution is shaped exclusively by clonal selection through competition. Of note, models of clonal selection and cooperation are not mutually exclusive and can complement each other. Yet, a key feature of clonal cooperation is that tumors benefit from clonal diversity because this allows collaborative interactions. This raises several questions: What factors mediate cooperativity? Can subclones be relevant also when present at low frequency? What prevents their further expansion? What is the role of the tumor microenvironment (TME)? At what stage of tumor progression, from premalignant disease to metastasis, is cooperation most relevant? What is the clinical relevance of clonal cooperativity, in particular in the context of treatment resistance?

Some excellent previous reviews have discussed several examples of clonal cooperativity [4, 14]. Here, we focus on key unanswered questions, identify overarching principles, and provide an outlook on future developments, suggesting where the field may head.

## 3 Clonal cooperativity: overarching principles

### 3.1 Secreted factors as key mediators of clonal cooperativity

Cooperation between subclones requires intercellular communication, which can happen through either direct cell–cell interactions or secreted molecules (Fig. 1). Remarkably, the vast majority of published cases of clonal cooperativity is mediated by secreted factors [5, 6, 7, 8, 9, 10, 11, 18, 19, 20, 21, 22, 23, 24, 25, 26]. For example, the facilitation of metastasis of NE cells by non‐NE cells in SCLC [18] is mediated by secreted FGF2 [21], the growth of *Ras* mutant clones is induced by secreted Wnt ligands in mammary tumors [7] or unpaired cytokines (related to IL‐6) in Drosophila models [12], and resistance to epidermal growth factor receptor (EGFR)‐targeted therapy in colorectal cancer [8], as well as metastasis of ovarian cancer [23], can be induced by amphiregulin secreted by a minor tumor cell subpopulation.

The role of secreted factors as mediators of clonal cooperation is expected, as secreted factors almost by definition affect neighboring cells. The extent to which secreted factors can affect other clones depends both on the degree of spatial intermixing among clones and the traveling distance of secreted factors. Multi‐region sequencing studies revealed that different subclones can grow in a genomically intermixed pattern [27, 28, 29, 30], which would facilitate interactions between different populations. Evidence for clonal mixing exists from spatial genomics studies as well [31], but more segregated growth of genetically distinct subclones has also been reported [32, 33]. When clones are more spatially confined, there is less potential for crosstalk through secreted factors that primarily act at short range. Secreted proteins, including growth factors (GFs) and Wnt ligands, can be sequestered by cell surface proteins or the extracellular matrix [34, 35], which limits their diffusion range, although they can still form concentration gradients through intercellular transport [36]. On the contrary, secreted factors can also signal at longer ranges. Using intravital imaging of mouse breast and ovarian cancers, Hoekstra et al. [37] showed that IFNγ can spread up to a distance of 800 μm and that long‐range effects of IFNγ can impair tumor growth of bystander cells after T‐cell pressure. Furthermore, secreted factors can reach the circulation and create premetastatic niches at sites distant from the primary tumor [5, 38]. Surprisingly, in an *in vivo* spatial functional genomics screen (Perturb‐map) for tumor‐promoting effects of secreted proteins in ovarian cancer, loss of *CCL7* promoted tumor growth, despite the presence of adjacent clones with normal *CCL7* expression. This was explained by the fact that, even though CCL7 is a secreted chemokine, its protein expression was highly localized [39]. More extensive investigation of the factors that determine the action radius of (subclonal) secreted factors will be important to determine their potential to mediate collaborative interactions between subclones.

Despite the frequent involvement of secreted factors in clonal cooperativity, the overall cancer‐intrinsic secretome is poorly defined in general, let alone to what extent it differs between subclones. Bulk sequencing approaches do not resolve whether secreted factors are derived from cancer cells, or from stromal or immune cells. Moreover, these studies typically rely on RNA expression, which correlates only modestly with protein levels [40, 41, 42], particularly for secreted proteins [41]. While single‐cell RNA sequencing approaches have the ability to identify cancer‐cell‐specific gene expression, the sparsity of reads further complicates the detection of secreted proteins which can be lowly expressed [39, 41].

To identify cooperation mechanisms mediated by secreted factors, there is a need to better define the cancer‐intrinsic secretome, and understand what factors are shared across all clones and which are unique to individual subclones. In mass spectrometry, lowly abundant secreted proteins are frequently overshadowed by highly abundant proteins, such as albumin. Recent advancements in click‐chemistry based techniques have revolutionized our ability to efficiently label secreted proteins by tumor cells. These developments enable for specific enrichment of secreted proteins, hence allowing high resolution detection of secreted proteins using mass spectrometry [43, 44]. Applying this method to isolated individual clones will allow defining the (sub‐)clonality of secreted factors. At this stage, this still requires short‐/long‐term culture to expand clones to sufficient levels for secretome analysis. However, single‐cell proteomics methods are on the rise [45], which paves the way for secretomics profiling at single‐cell resolution [46, 47, 48].

Most evidence exists for clonal cooperation through secreted proteins directly secreted into the TME. Nonetheless, other forms of secreted mediators are found in the form of metabolites [19, 49, 50] and extracellular vesicles [22, 26]. Extracellular vesicles have been shown to non‐cell‐autonomously induce epithelial‐to‐mesenchymal transition (EMT) and promote metastasis in breast cancer through transfer of miRNA200 [22], and to enhance several malignant phenotypes (cell growth, invasion, and adhesion) when released from ganglioside positive to ganglioside negative melanoma cells [26].

In addition to different forms of secreted mediators, there are also examples of cooperativity through direct cell–cell contact [51, 52, 53, 54, 55, 56] (Fig. 1). For example, physical transfer of organelles has been shown to promote cancer survival and growth. Saito et al. [54] found that mitochondrial exchange from bone marrow mesenchymal stem cells to acute myeloid leukemia cells resulted in increased adaptation and resistance to oxidative phosphorylation inhibition. Furthermore, migrasomes (vesicular organelles formed on retraction fibers behind migrating cells) have been found to transfer PTEN mRNA from PTEN‐proficient to PTEN‐deficient tumor cells, leading to reduced AKT phosphorylation and ultimately inhibiting cell proliferation [56]. However, as mitochondria exchange is so far only found between nonmalignant and malignant cells [57], and as migrasome exchange has not been shown to have cooperative effects, the role of organelle exchange in clonal cooperativity remains an underexplored topic. Another form of direct cell–cell contact mediating cooperativity are ligand–receptor interactions. For example, expression of the inhibitory cell surface molecule PD‐L1 on a subset of melanoma cells can protect other cells not expressing that molecule from T‐cell attack [55]. Furthermore, microtubule networks have been described in astrocytic brain tumors [53], which connect cancer cells and increase their growth, invasion, and resistance capacity. In conclusion, while future research may identify additional layers of intercellular cooperation, the current evidence points to a major role for secreted proteins as a key mediator of clonal cooperation (Table 1).

**Table 1:** Secreted factors in clonal cooperation. CM, conditioned medium; EMT, epithelial‐to‐mesenchymal transition; EVs, extracellular vesicles; NE, neuroendocrine; TFs, transcription factors; wt, wild‐type.

Type	Secreted factor	Secreting cell type	Affected cell type	Effect	Cancer type	Study
Proteins	Unpaired cytokines (related to IL6)	*RAS*V12/*Scrib*‐clone mixture	*RAS*V12/*Scrib*‐clone mixture	Growth, invasion	Drosophila eye‐antennal disks	Wu et al. (2010) [12]
Proteins	IL6, LIF	*EGFR‐*mutated cancer cells	*EGFR*wt cancer cells	Growth	Glioblastoma	Inda et al. (2010) [9]
Proteins	Cytokines, chemokines (CCL2, CCL5, CCL9, CXCL1, CXCL5, Selp, sTNFR1)	Non‐NE cancer cells	NE cancer cells	Growth, metastasis	Lung (SCLC)	Calbo et al. (2011) [18]
Proteins	CM: progranulin	Therapy‐resistant cancer cells	Therapy‐sensitive cancer cells	(Chemo) resistance	Colorectal	Bose et al. (2011) [5]
Proteins	Wnt	*Hras*wt *Wnt1* high cancer cells	*Hras*wt *Wnt1* high cancer cells	Growth	Breast	Cleary et al. (2014) [7]
Proteins	TGF‐α, amphiregulin	Therapy‐resistant colorectal cancer cells	Therapy‐sensitive colorectal cancer cells	(EGFRi) Resistance	Colorectal	Hobor et al. (2014) [8]
Proteins	IL11	IL11 overexpressing cancer cells	Non‐IL11 overexpressing cancer cells (same cell line)	Growth	Breast	Marusyk et al. (2014) [11]
Proteins	Fgf2	Non‐NE cancer cells	NE cancer cells	Metastasis	Lung (SCLC)	Kwon et al. (2015) [21]
Proteins	TFs: Six1, Snail1, Twist1	EMT cancer cells	Non‐EMT breast cancer cells	Metastasis	Breast	Neelakantan et al. (2017) [94]
Proteins	CM; CXCL2	*KMT5B*mut cancer cells	*KMT5B*wt cancer cells (other clones same cell line)	Invasion, migration	Pediatric glioblastoma, diffuse intrinsic pontine glioma	Vinci et al. (2018) [60]
Proteins	IL11, FIGF	IL11/FIGF overexpressing cancer cells	Non‐IL11/FIGF overexpressing cancer cells (same cell line)	Metastasis	Breast	Janiszewska et al. (2019) [10]
Proteins	Amphiregulin	Nontumorigenic cancer cells	Tumorigenic cancer cells (same cell line)	Metastasis	Ovarian	Naffar‐Abu Amara et al. (2020) [23]
Proteins	TGF‐β	Metastatic AKTP cancer cells	Nonmetastatic AP cancer cells	Metastasis	Colorectal	Kok et al. (2021) [72]
Proteins	LDHA, DKK3	*Myc* driven (amplified) cancer cells	Non‐*Myc* driven cancer cells	Metastasis, angiogenesis	Medulloblastoma	Qin et al. (2022) [25]
Proteins	CM; TGF‐β1, proteases	Highly invasive cancer cells	Lowly invasive cancer cells	Metastasis	Breast, Lung	Carneiro et al. (2023) [6]
Proteins/metabolites	CM	Therapy‐sensitive cancer cells	Therapy‐resistant cancer cells	Growth, metastasis, resistance	Melanoma, lung	Obenauf et al. (2015) [24]
Metabolites	CM: metabolite fraction	Clonal cancer cells	Clonal cancer cells (same cell line)	Growth, metastasis	Breast	Hershey et al. (2023) [19]
Metabolites	CM: metabolite fraction	CMS1 cancer cells	CMS4 cancer cells	(Chemo) Resistance, invasion	Colorectal	Kallberg et al. (2023) [20]
Metabolites	Aminopeptidases (CNDP2)	Aminopeptidase secreting cancer cells	Non‐aminopeptidase secreting cancer cells	Growth	Melanoma, breast, lung (NSCLC), colorectal, pancreas	Guzelsoy et al. (2025) [49]
Metabolites	Lactate, MCT4, MCT1	Non‐NE cancer cells	NE cancer cells	Growth (metabolism)	Lung (SCLC)	Peinado et al. (2025) [50]
EVs	Extracellular vesicles (miR200)	Metastatic cancer cells	Nonmetastatic cancer cells	Metastasis	Breast	Le et al. (2014) [22]
EVs	Extracellular vesicles	Ganglioside expressing cancer cells	Ganglioside negative cancer cells	Growth, invasion, cell adhesion, oncogenic signaling	Melanoma	Yesmin et al. (2023) [26]

### 3.2 Minor subpopulations can drive tumor growth

It may be intuitive to focus our attention on the dominant clone in the tumor, but subclones can profoundly affect the overall phenotype, behavior, and therapeutic response of the tumor even when making up (very) small proportions of the tumor. In a mouse model of SCLC, non‐NE cells provide essential trophic support to faster growing NE cells, with non‐NE cells being stably maintained at 25% of the population [58]. Human glioblastomas frequently contain subclones with different *EGFR* aberrations [59]. Remarkably, experiments in which mixtures of human *EGFRwt* and *EGFRvIII* clones were co‐injected into mice showed that the *EGFRvIII* clone can be as rare as 1% and still exert a significant effect on proliferation of *EGFRwt* clones [9]. Similarly, even though only 1–3% of tumor cells expressed IL‐4 in a mouse ovarian cancer model, its knockout still profoundly affected the composition of the TME and sensitivity to anti‐PD1 therapy [39]. Likewise, in a study of human diffuse intrinsic pontine glioma (DIPG), a small (< 1%) subclone was identified that contained a mutation in the epigenetic modifier *KMT5B*. This clone promoted the invasion and migration of nonmutant admixed cells in a cooperative manner in primary DIPG cell cultures [60]. Interestingly, a pan‐cancer analysis of recurrent subclonal mutations showed an enrichment for epigenetic modifiers [1]. It will be of interest to determine whether subclones carrying mutations in epigenetic modifiers show potential for collaborative interactions beyond this single case.

An important question related to the presence of such relevant minor clones is why they do not reach clonal dominance. Different factors have been shown to underlie this. First of all, spatial constraints limit clonal expansion [61], and clones with similar fitness arising simultaneously can stabilize each other (clonal interference) [11]. Furthermore, the role that a subpopulation fulfills can come at a fitness cost. For example, clones with mitochondrial defects enter cell cycle arrest, but can simultaneously promote the invasiveness of neighboring clones [17]. Vice versa, NE SCLC cells can be electrically active, which directly promotes their malignancy. However, this comes with a high energy demand, which is provided by non‐NE cancer cells, and therefore, neither population will become dominant [50]. Ultimately, whether a subclone will go extinct, become dominant, or stabilizes as a minor population, depends on the balance between its fitness benefit and fitness cost. For example, the presence of exogeneous GFs (e.g., from stromal cells) can obviate the need for a subclone to provide these, shifting the cost/benefit ratio and causing their extinction [62]. In addition, clonal dynamics are shaped by interdependencies between subclones: as for example shown by the Drosophila studies discussed in Section 2, when two populations divide labor in such a way that they need each other to grow or metastasize, neither will reach clonal dominance [12, 18]. Finally, a clone can stay small if it provides support to another clone that proliferates faster. In extreme cases, it may get outcompeted, resulting in tumor collapse [11].

Clearly, small clones matter, and targeting them can significantly suppress tumor growth [9, 63]. Since some clones can profoundly affect tumor behavior even when representing as little as 1% of the tumor mass [9], this limits their detection using standard sequencing approaches [64]. It will be important to define which mutations are relevant when subclonal, as well as the minimal proportion at which they still significantly affect tumor behavior, to determine in which cases deeper sequencing may be warranted. The ultimate aim of such efforts will be to widen the arsenal of therapeutic options by targeting currently ignored low‐frequency targets with strong non‐cell‐autonomous effects.

### 3.3 Nonheritable heterogeneity as a source of cooperation

In addition to genetic or epigenetic heritable factors, ITH is also driven by nonheritable factors that result in phenotypically diverse subpopulations. Indeed, cancer cells with the same genetic background can show diverse phenotypes, depending on the signals they receive from their microenvironment [65, 66]. Spatial and temporal differences in, for example, nutrient availability, GF abundance, oxygen levels, surrounding matrix, and the presence of stromal and immune cells can induce vast phenotypic heterogeneity [67, 68]. Nongenetic phenotypic heterogeneity can also arise independent of the microenvironment through self‐organizing principles in tumors. This has for example been observed in the generation of non‐NE cells from NE progenitors to retain a balance between both populations [58], or in the dynamic maintenance of Wnt‐sending and Wnt‐receiving cell populations in pancreatic cancer [69].

These more dynamic sources of heterogeneity are important additional substrates for division of labor and cooperation [49, 58, 69]. However, as formally showing cooperation requires comparing the behavior of individual versus mixed populations, this typically involves isolating specific populations which need to retain their phenotype at least for the duration of the assay. Therefore, most research on clonal cooperation is focused on differences between subpopulations that are relatively ‘fixed’ [8, 9, 12, 18, 23]. Alternative approaches will be needed to investigate the impact of cooperation between subpopulations with different phenotypes, independent of clonal ancestry (see Section 5). Advances in spatial sequencing and imaging technologies will allow defining both genotype and phenotype of cancer cells [33, 70, 71]. This can be combined with *in vivo* studies where specific subpopulations are eliminated through inducible suicide switches to determine their non‐cell‐autonomous tumor‐promoting roles [69]. Such studies will be important to determine the relative importance of heritable versus nonheritable heterogeneity in driving cooperation between diverse subpopulations.

### 3.4 The tumor microenvironment as a cooperating partner

Aside from its role in shaping the phenotypic heterogeneity of cancer cells, the TME also acts as a ‘partner in crime’: cooperation can occur directly between cancer subpopulations, but can also involve the modulation of nonmalignant cells in the TME (Fig. 1). Examples of this include the generation of a pro‐metastatic niche by stimulating neutrophils through IL11 secretion [10], and suppression of CD8^+^ T cells by subclonal PD‐L1 expression [55]. Moreover, Kok et al. [72] show that a metastatic colorectal cancer clone induces the generation of a fibrotic niche, likely through activation of hematopoietic stem cells, and thereby promotes the colonization of a different nonmetastatic clone. *In vivo* knockout of *Tgfbr2* significantly reduced metastasis formation, showing that subclonal TGF‐β secretion was involved in creating this fibrotic niche [72].

The investigation of clonal cooperation in the context of the TME is difficult in *in vitro* and *ex vivo* culture systems. However, recent advances in co‐cultures of organoids with immune cells or stromal cells make it possible to functionally assess the interplay between different cell types [73, 74]. Organoids can be used to isolate individual clones [75] that can subsequently be co‐cultured with immune cells [76] to determine the impact of individual clones versus combinations thereof. Such reductionist models allow zooming in on communication axes of interest, but lack the broader context of the TME. To more inclusively capture the effect of the whole microenvironment, mixtures of (barcoded) clones can be transplanted into *in vivo* mouse models [11, 77]. However, experimental models that allow both the dissection of ITH and incorporation of a human TME are still lacking. This makes it important to consider the limitations of such model systems, and ultimately, determining the influence of the TME on clonal cooperativity will require a combination of both reductionist and more holistic model systems.

### 3.5 Clonal cooperativity from premalignancy to metastatic disease

Different levels of ITH are found at different stages of disease [78], raising the question of what role clonal cooperativity plays at different stages of tumor progression, from premalignancy to metastatic disease.

Recent studies show that clonal cooperativity plays a prominent role in early stages of tumorigenesis. In colorectal adenomas, a high level of heterogeneity and subclonality of driver mutations is found [79]. For example, *KRAS* and *APC* mutations were heterogeneous between crypts in early colorectal tumors [80]. This finding challenges the model that tumors arise from a single clone and highlights the opportunity for cooperation between different clones in early tumor formation. Indeed, a recent lineage tracing study shows that many colorectal adenomas have a polyclonal origin, often consisting of a major clone enriched in Ras signaling and a minor clone with a Myc/stemness signature [81]. Interestingly, polyclonal tumors grew faster than monoclonal tumors, although this study did not establish whether the increased growth was the cause or consequence of polyclonality. Similarly, Islam et al. [82] coupled single‐cell barcode tracking in mouse adenomas to single‐cell profiling of human precancers to show the frequent polyclonal origin of colorectal precancers. By mapping single‐cell phylogenies throughout intestinal tumorigenesis, Lu et al. [83] predicted multiple receptor–ligand interactions between different cancer cell subpopulations in precancer. Importantly, they showed that progression from precancer to more advanced cancers is associated with a ‘polyclonal‐to‐monoclonal’ transition and a significant loss of receptor‐ligand interactions between epithelial subpopulations [83].

If early tumor formation is characterized by the interplay between different clones, it becomes relevant to consider the local environment in which precancerous mutations arise. ‘Healthy’ tissues are riddled with mutations and form a mosaic of different clones [84, 85, 86, 87]. This raises the possibility that the likelihood for a mutated clone to form a tumor is influenced not only by its own genetic background, but also by that of its neighbors. Indeed, the Drosophila studies cited above (see Section 2) [12, 17] show that the ability of mutant cells to grow out is affected by the specific signals they receive from neighboring cells carrying different mutations. Interestingly, *NOTCH* genes are frequently mutated in normal skin [85, 86], and *Notch1* mutations have been shown to promote skin tumor formation (also of *Notch1* wild‐type cells) in a non‐cell‐autonomous manner [88]. This concept has however not been explored in‐depth in mammalian tissues and remains at this stage largely hypothetical.

Despite a reduction in clonal diversity in the transition from precancer to cancer [78, 83], once the clone that seeds the primary tumor starts to expand, this is accompanied by the accumulation of extensive ITH [1, 2, 78]. Clonal cooperativity is often described in the context of primary cancers [9, 18, 50, 58, 60]. As a tumor grows, the more specialized tasks will need to be carried out as it can rely less on support from host cells. Indeed, in several described cases of clonal cooperation, certain subclones provide such a specialized function from which other clones benefit. This includes autocrine production of GFs [62, 89], and induction of angiogenesis to adapt to low oxygen [10]. As an additional illustrative case, Peinado et al. [50] report that early‐stage SCLC is strongly innervated, but this decreases in late‐stage tumors, when NE cells start to express neuronal markers themselves. The electrical excitability of NE cells promotes malignancy, while making them dependent on non‐NE cells for trophic support, indicating functional diversification and task division coinciding with tumor progression and increased ITH.

Heterogeneity of primary tumors has been correlated with a high rate of metastasis [90]. Clonal cooperation has been shown to promote metastasis in multiple ways, in line with its multi‐step nature. Cooperation where a subpopulation increases the metastatic potential of another subpopulation has been found in the form of breaking through the extracellular matrix [91, 92, 93], induction of EMT [6, 22, 94], immunomodulation [10], and formation of the premetastatic niche [72]. For example, Chapman et al. [91] showed in heterogeneous zebrafish xenograft models that invasive melanoma cells produce protease MT1‐MMP to degrade the ECM, enabling co‐invasion of noninherently invasive cells. Metastasis is traditionally viewed as a bottlenecking event that involves selection of the clone most fit to break out, travel through the bloodstream and settle in a new microenvironment. However, polyclonal metastasis is not uncommon [95, 96], and at the time of clinical detection, metastases consist of millions of cells with the potential for diversification at every cell division. Thus, although metastatic lesions are generally found to be less heterogeneous than primary tumors [78], there still is considerable ITH [78, 95]. Anticancer treatments can lead to the elimination of certain clones, resulting in reduced heterogeneity, although the extent of this is highly context‐dependent and even good clinical responses do not always result in extensive loss of genetic diversity [97, 98, 99]. Importantly, therapeutic pressure can also permit latent populations that were previously only present as minor subpopulations to grow out [100, 101, 102]. For example, in PDX models of triple negative breast cancer, cisplatin resulted in the outgrowth of initially chemotherapy‐resistant subclones with initially low fitness [102]. This indicates that clonal diversity in metastases can provide a substrate for cooperation in the defense against systemic anticancer therapies.

The extent of ITH therefore is not fixed, but changes over time and in different tumor stages. This poses a challenge for the identification of crucial clonal interactions. For example, if a clone that is necessary for the onset of metastasis later gets outcompeted, it will not persist in samples taken from the metastatic lesion [18, 23]. Therefore, many important interactions can remain unobserved. Establishment of functional models directly derived from patient samples, such as patient‐derived organoids or PDX models [102, 103], at different stages of cancer—from premalignancy to metastatic disease—will provide opportunities to functionally interrogate clonal interactions at different time points.

### 3.6 Clonal cooperativity in the context of treatment resistance

Despite advances in understanding mechanisms of tumorigenesis and the development of new anticancer therapies, many cancers still acquire resistance and relapse [68]. Heterogeneous tumors respond particularly poorly to therapy [3], with pre‐existing heterogeneity as well as diversification during therapy allowing for the outgrowth of specific clones [102]. Indeed, different clones from the same tumor have been shown to respond differently to chemotherapies and targeted agents [75]. While therapy resistance has historically largely been approached from a cell‐autonomous perspective, there is evidence that therapy‐resistant and therapy‐sensitive clones can cooperate to drive resistance of the tumor.

Bose et al. [5] argue that clonal cooperation can explain why a relatively large percentage of cells remain after chemotherapy, whereas intrinsically resistant cells only comprise a low percentage of the original tumor. They found that oxaliplatin‐resistant (OxR) colorectal cancer cells secrete factors that stimulate growth of chemonaive cells and render them less sensitive to oxaliplatin treatment. Interestingly, they observe that tumors with mixed OxR parental cells grew larger than tumors composed of 100% of OxR cells, a finding that would be interesting to explore further, since this indicates that the sensitive cells serve another purpose in the outgrowth of tumors under chemotherapeutic pressure, or that the acquisition of a resistance program comes with a fitness cost.

Resistant subpopulations endowing resistance on an intrinsically sensitive subpopulation is also found for targeted therapy. Mutations in *KRAS* are a common resistance mechanism to cetuximab (anti‐EGFR) therapy in colorectal cancer. Surprisingly, the fraction of *KRAS* mutant cells in tumors that acquired resistance to cetuximab is often relatively low [101]. Hobor et al. [8] found that resistant cells express higher levels of the secreted proteins TGF‐α and amphiregulin. When added to sensitive cells, these factors sustained ERK signaling downstream of EGFR and thereby protected these cells from EGFR‐targeting cetuximab treatment. Importantly, sensitive cells alone did not grow out under cetuximab pressure, but co‐culture of resistant and sensitive cells in the presence of cetuximab resulted in the outgrowth of both cell populations, indicating that resistant cells protected sensitive cells from treatment. Also in the context of targeted therapy, Obenauf et al. [24] found a mechanism through which melanoma cells sensitive to therapies targeting kinase inhibitors support resistant cells. They showed that targeted therapy altered the secretome of therapy‐sensitive cells that not only supported their own survival, but also promoted outgrowth of mutated resistant cells.

In the context of immunotherapy, Williams et al. [55] found clonal cooperation between *Ifngr2*‐ or *Jak1*‐mutant and wild‐type clones. In a pooled, genome‐wide CRISPR screen, these mutant clones grew out successfully *in vitro* in the presence of PD‐L1 targeting immunotherapy, suggesting that these mutations can confer immunotherapy resistance. Surprisingly however, tumors fully consisting of these resistant cells were found to be better controlled immunologically *in vivo*, since these mutations resulted in a decreased upregulation of PD‐L1. This contrasted with the setting of the polyclonal CRISPR screen where each clone has a different knockout. Indeed, in a mixed transplant with both wild‐type and mutant cells, PD‐L1 expression of wild‐type cells ultimately inhibited immune cells sufficiently to enable mutant cells to grow out. This indicates that clones can vary in their response to therapy depending on whether there is potential for cooperation.

As shown by these examples, clonal cooperation can act in different ways to increase the resistance of the tumor to different types of anticancer treatments. However, it is unclear whether these examples are anecdotal, or have relevance for therapy resistance in a broader context. Functional studies that determine the extent to which therapy resistance is driven by cell‐autonomous versus non‐cell‐autonomous mechanisms are needed to determine the clinical relevance of clonal cooperativity. It is also relevant to assess to what extent post‐treatment samples still contain therapy‐sensitive clones, as was observed for *KRAS* wild‐type cells in cetuximab‐resistant tumors [101]. To this end, pre‐ and post‐treatment biopsies are valuable, especially if they can subsequently be used for *in vitro* models to functionally assess the treatment sensitivity of different subclones. Identifying the extent to which therapy resistance is non‐cell‐autonomous and driven by cooperative interactions will be highly relevant to design future treatment approaches (see Section 4).

## 4 Therapeutic opportunities of targeting clonal cooperativity

Clonal cooperativity has several implications for anticancer therapy. Traditional (targeted) therapies are directed specifically at the clone containing the targetable alteration. Given the extensive heterogeneity in tumors, this will almost inevitably result in the selection of a therapy‐resistant subclone. In contrast, targeting clonal cooperation is focused on non‐cell‐autonomous effects and therefore multiple populations, regardless of whether they are specifically targeted or not, can be affected by eliminating their connecting link: ‘killing many birds with one stone’. The ultimate goal of identifying clonal cooperativity is to find and target the weakest link of a heterogeneous tumor to induce its fall. This rationale is illustrated by a study that showed that pancreatic ductal adenocarcinomas consist of different populations of Wnt‐secreting (Wnt‐S) and Wnt‐responding (Wnt‐R) cells. *In vivo* ablation of Wnt‐S cells significantly reduced both cell types and strongly suppressed tumor growth, regardless of the fact that Wnt‐R cells were not directly targeted themselves [69].

Secondly, subclones may decrease therapy efficiency by casting a ‘protective shield’ over populations that would otherwise be sensitive. This is most clearly illustrated by work that shows that anti‐EGFR‐therapy‐resistant subclones can protect sensitive cells through the secretion of EGFR ligands [8]. It will be relevant to test experimentally whether targeting such protective factors can resensitise the population that benefits from paracrine protection.

Third, given that minor subclones can exert profound effects on the tumor, this suggests that therapeutic targeting should not necessarily be limited to targets that are clonal. For example, a minor (1%) mutated (dEGFR) cell population in glioblastoma multiforme tumors has been shown to increase growth of EGFR amplified (EGFRvIII) cells through secretion of IL6 and leukemia inhibitory factor (LIF). Knockdown of IL6, LIF or their receptor gp130 abolished this growth enhancement [9]. Morrow et al. [63] also identified IL6 as subclonally secreted factor that non‐cell‐autonomously promoted growth in breast cancer. Importantly, this effect was blocked by the clinically approved IL6 receptor blocking antibody tocilizumab, pointing toward a possible strategy to target this cooperative mechanism in breast cancer patients.

Finally, an appealing therapeutic approach that is particularly relevant in the context of clonal cooperativity is ‘evolutionary steering’. Subclones that are crucial for the maintenance of the tumor through non‐cell‐autonomous effects may carry a fitness cost, which could be exploited to drive a tumor toward extinction. For example, in a mouse xenograft model of a breast cancer line, Marusyk et al. [11] showed that an IL11‐secreting subpopulation drove tumor growth through increased vascularization and remodeling of the extracellular matrix, but was outcompeted by faster proliferating competitors that ultimately could not continue growing without the presence of the IL11‐secreting population. This provides a basis for the design of therapies that induce a similar dynamic by manipulating cost/benefit ratios of the production of public goods. For example, Archetti [104] shows that when production of GFs is associated with a fitness cost, producer cells can get outcompeted by defector cells with GF knockouts when exogenous GF levels are sufficiently high, ultimately resulting in reduced tumor growth.

It should be noted that actual efforts to directly assess the efficacy of targeting clonal cooperativity in (pre‐)clinical models have so far been limited. For the field to move forward, it will therefore be important not only to identify where, when, and how clonal cooperativity occurs, but also what the effect of therapeutic interventions is. This will reveal potential challenges of translating currently ongoing research into clinical application. We envision at least two complexities that will need to be addressed. First, while eliminating a key node in the tumor can take other populations down in its fall, it may also induce adaptive responses that give space to new cooperative interactions. Therefore, it will be essential to assess these adaptive responses during therapeutic intervention, for example by longitudinal sequencing. Targeting of clonal interdependencies could also be combined with treatments that minimize ITH to limit the potential for new interactions. Combination with therapies aimed at the biggest bulk of the tumor to induce rapid reduction of tumor load will improve patients' quality of life, reduce ITH, and limit emergence of resistance mechanisms.

Second, targeting essential (growth) factors is challenging, since these factors are often also essential for normal cells; many signaling pathways that are involved in clonal cooperation are also associated with normal physiological processes [6]. For example, targeting Wnt, a secreted factor that can non‐cell‐autonomously promote tumor growth, has proven challenging due to harmful side effects to homeostatic tissue repair and normal stem cells [105]. Therefore, the most successful interventions should target dependencies that are unique to the TME, such as cooperative nutrient scavenging that is particularly important under the amino acid deprived conditions in a tumor [49].

## 5 Technological advances

There is a substantial scientific basis for the existence of clonal cooperativity, with evidence from multiple model systems in different cancer types (Table 1). At the same time, an important research gap remains, in particular when it comes to understanding the extent and impact of cooperativity in actual patients with cancer. This gap is largely due to technological limitations. Here, we highlight several recent technological advances that offer new opportunities to study clonal cooperativity, in particular in contexts that allow better capturing naturally occurring heterogeneity.

The vast number of spatial and single‐cell RNA sequencing studies allow for the identification of crosstalk between subpopulations in patient tumors based on expression levels of known ligand–receptor pairs. To this end, various computational tools (e.g., CellPhoneDB [106] and CellChat [107]) have been developed and are continuously being improved [108, 109, 110].

So far, ligand–receptor‐based models have mostly been used to infer communication between tumor cells and other cell types (e.g., immune cells [111, 112] or stromal cells [113, 114]), which is interesting to use for studying the TME as a cooperating partner. However, it will be necessary to also apply these methods to tumor cell—tumor cell interactions. For example, Lu et al. [83] used CellChat to infer interactions between epithelial subpopulations in normal colon, early polyclonal and late monoclonal cancer lesions, finding a decrease in communication as the tumor progressed.

An important limitation of these ligand–receptor computation tools is that their predictions are restricted to defined sets of receptors and ligands and therefore do not offer a completely unbiased approach. Moreover, they generate a list of potential interactions without information on which interactions are most relevant. This can be improved to some extent using computational approaches, such as ContactTracing, which exploits the variability in single‐cell sequencing data to predict the cellular response to a particular ligand–receptor interaction [115]. Moreover, incorporation of spatial information will increase the ability to identify interacting subpopulations [116]. However, ultimately, *in vitro* or *in vivo* validation will be essential to prove that these interactions actually drive cooperative behavior.

Genetic barcoding methods allow tracking clonal dynamics over time and in response to perturbations *in vitro* and *in vivo* [82, 117, 118, 119]. Whiting et al. [120] combined genetic lineage tracing and population size data to infer drug resistance dynamics; in different colorectal cancer cell lines, they identify either outgrowth of a pre‐existing resistant subpopulation or phenotypic switching as the origin of resistance. Identifying which populations give rise to (polyclonal) early tumors [82, 121] or metastasis [122] and which are responsible for treatment resistance [120, 123] can point toward essential subpopulations during these bottlenecking events. With recent advances in self‐guiding CRISPR gRNAs, it is possible to label clones *in situ*, providing a comprehensive view of clonal heterogeneity in its native composition and TME [82, 124].

Although clonal barcoding can capture heterogeneity in *in vivo* models, it is not applicable to longitudinal tracking of human patient tumors. As an alternative, circulating tumor DNA (ctDNA) or cell free DNA (cfDNA) sequencing can be used, using a noninvasive sampling method and measuring changes in clonal composition over time [125, 126, 127]. For example, Williams et al. [127] collected cfDNA from ovarian cancer patients before, during, and after treatment to identify that drug resistance is frequently polyclonal.

Monitoring clonal dynamics *in vivo* is also supported by advanced imaging technologies. Fluorescent barcoding enables the investigation of clonal composition and dynamics in a spatial context. Where genetic barcoding methods ultimately still require sample digestion to retrieve clonal information, fluorescent barcoding allows for the direct assessment of clonal make‐up in their spatial context [128, 129]. Importantly, recent developments in imaging technologies allow for live imaging to follow clones *in situ* over time in (genetically engineered) organoids [130] and *in vivo* models [131, 132]. For example, Heinz et al. [132] use intravital microscopy of mouse livers to study the relative presence of cancer stem cells and noncancer stem cells during *in vivo* metastasis formation and outgrowth.

Furthermore, imaging methods to track secreted factor expression and spread are especially relevant in the context of clonal cooperation, since these factors almost by definition have a non‐cell‐autonomous effect. Mollaoglu et al. [39] use Perturb‐map to knock out several target genes encoding secreted factors and simultaneously incorporate unique protein barcodes to map these different clones with multiplex imaging. In addition, the use of biosensors allows defining the spatiotemporal spread of cytokines, such as IFNγ [37].

As discussed above (Section 3.4), organoid and PDX models are uniquely suited to capture the naturally occurring heterogeneity of human cancers, which allows isolating clones that can then be reintroduced into different combinations [78, 79]. Alternatively, more untouched strategies to functionally assess cooperation can be used that do not require the upfront isolation of specific clones. Using conditional suicide genes, specific subpopulations can be eliminated *in vivo* and therefore in the same TME [39, 133]. For example, Tammela et al. [133] show that Wnt‐secreting and Wnt‐responsive subpopulations cooperate by eliminating the Wnt‐secreting cells. An important advantage of *in vivo* elimination of clones or subpopulations is that it can be applied to models with endogenous tumors, which have an advantage over PDX models in recapitulating heterogeneity within the native TME. Clonal retrieval, the identification, and recovery of specific clones from a mixed population of cells, allows on the other hand the investigation of (specific) combinations of clones [119, 124, 134, 135, 136, 137]. For example, Umkehrer et al. [119] developed CaTCH, a method to trace and isolate clones for functional experiments. They expanded and re‐injected RAFi/MEKi treatment‐naïve and resistant clonal pairs into their mouse model to assess subclone evolution and the origin of resistance. Assessment of tumor behavior (e.g., growth and metastasis formation) with different clonal compositions is essential in proving the presence and relevance of clonal cooperation, making both elimination and isolation of barcoded clones valuable approaches.

## 6 Concluding remarks

**Fig. 2:**
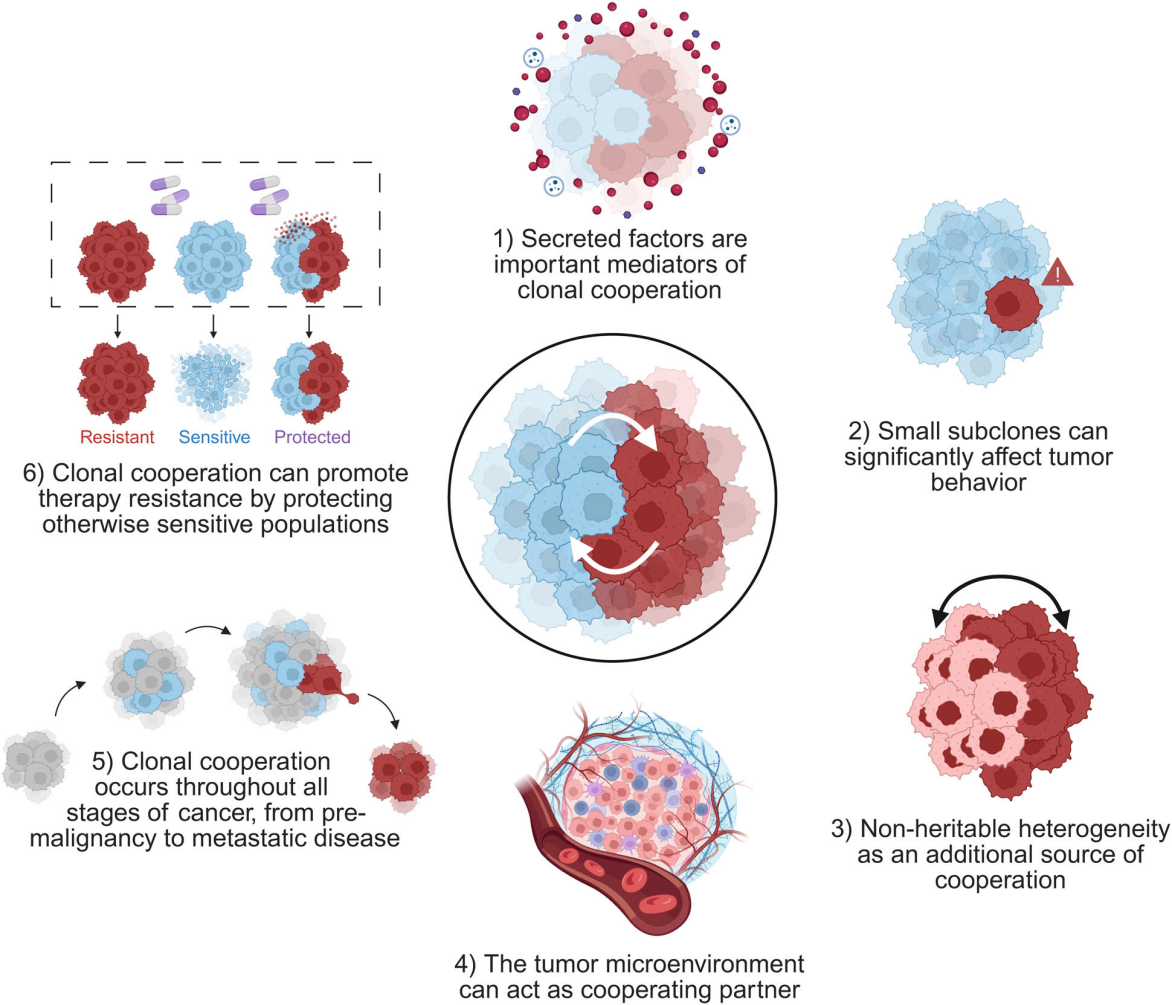
Overarching principles of clonal cooperation. Figure created using BioRender.com.

We have reviewed the available data on clonal cooperativity in heterogeneous cancers and found evidence for this concept in diverse settings. Reinterpreting these data have allowed us to extract six common themes that can serve as focus points for further investigation (Fig. 2).

First, secreted factors are found to be key mediators of clonal cooperation, highlighting the importance of deeply characterizing the currently poorly defined cancer cell‐intrinsic secretome, particularly at single clone resolution. Second, tumor cell subpopulations can profoundly affect tumor behavior even when making up (very) small proportions of the tumor. In some cases, cooperative interactions explain why some mutations remain subclonal, such as for *EGFRvIII* in glioblastoma [9], or *KRAS* mutant clones in colorectal cancer [101]. Third, there is considerable nonheritable heterogeneity, either induced by the TME or through self‐organizing cellular hierarchies [58, 69, 70]. Yet, most research on cooperation between cancer subpopulations has focused on heritable heterogeneity. It will be essential to dissect the contribution of both heritable versus non‐heritable heterogeneity on cooperative interactions. The rapid advance of spatial sequencing and imaging technologies can inform how cancer cell phenotypes are shaped by the microenvironment. In addition, *in vivo* models in which specific populations can be selectively eliminated will allow determining to what extent tumors depend on (nongenetically defined) subpopulations. Fourth, cooperation can involve modulation of nonmalignant cells in the TME and a combination of *in vitro* co‐culture models and *in vivo* transplantation of barcoded clonal mixtures will be needed to assess the interplay between different cell types. Fifth, clonal cooperation can play a role throughout all stages of cancer and is most relevant when diversity is large (such as in precancers before the escape of a strong driver subclone, large primary tumors, or extensive metastatic disease). Finally, clonal cooperation in the context of anticancer treatment can lead to the protection of otherwise sensitive subclones. However, a more systematic approach is needed to determine the extent to which resistant subclones can provide protection through non‐cell‐autonomous resistance mechanisms (Table 2).

**Table 2:** Open questions and experimental approaches to determine the impact of clonal cooperation.

What is the impact of clonal cooperation in human cancers?		
	Open questions	Experimental approaches
Secreted factors	What defines the cancer cell‐intrinsic secretome? To what extent does the secretome diverge between subclones? What is the traveling distance of subclonal secreted factors?	Bioorthogonal labeling methods for high resolution secretomics Isolation and expansion of individual clones for subclone‐specific secretomics Single‐cell proteomics/secretomics Spatial profiling of tissues; plasma proteomics
Minor subpopulations	Are recurrent subclonal mutations mediators of clonal cooperation? What is the lowest proportion at which a subclone can still significantly affect tumor function?	Characterization of the non‐cell‐autonomous effects of subclonal mutations Mixing experiments of different clones *in vitro* and *in vivo* Deep sequencing to identify low‐frequency mutations
Non‐heritable heterogeneity	How are cancer cell phenotypes defined by their local microenvironment? What is the relative importance of heritable versus nonheritable heterogeneity for cooperation between subpopulations?	Spatial profiling to determine the relation of cancer subclones or subpopulations to their local microenvironment *In vivo* elimination of specific subpopulations to determine their non‐cell‐autonomous tumor‐promoting role
The tumor microenvironment	How does the interplay between tumor cells and their microenvironment contribute to clonal cooperation?	*In vitro* co‐cultures of (subclonal) organoids with immune or stromal cells *In vivo* transplantation of barcoded clonal mixtures
Stages of disease	Is polyclonal tumor formation advantageous due to clonal cooperation? Do certain (combinations of) mutations in healthy tissue support tumor formation by providing cooperating signals? How does the clonal composition of a tumor change throughout the stages of cancer, and how does that impact clonal cooperativity?	Characterization of paired, longitudinal samples of primary tumors, recurrences, and metastases Isolation, expansion and barcoding of individual subclones at different stages of disease Establishment of clonally mixed tumor models *ex vivo* (organoids) and *in vivo* (PDX)
Treatment resistance	To what extent is therapy resistance driven by non‐cell‐autonomous mechanisms? To what extent are therapy‐sensitive clones still present in relapsed/resistant tumors? Are sensitive populations maintained in a relapsing tumor because they support resistant populations (division of labor)? Can the elimination of (minor) driver subclones induce tumor collapse?	Isolation of resistant subclones and functional assessment of their potential to drive resistance in a non‐cell‐autonomous manner Investigation of the presence of therapy‐sensitive and therapy‐resistant subpopulations in pre‐ and post‐treatment biopsies Identification and targeting of (recurrent) mediators of clonal cooperation

While the available literature suggests that clonal cooperation can have a profound impact on tumor behavior, it is important to emphasize that models of cooperation and competition are not mutually exclusive, and likely co‐occur. Clonal cooperation points at an additional layer of complexity that can help explain observations that are difficult to reconcile with models treating subclones as independent actors. For the field to move forward, it is now important to move from anecdotal reports to determining the overall relevance of this concept. The current state‐of‐the‐art shows that clonal cooperativity can happen, but how often does it happen? What is the extent of its effect? This will be particularly important in the context of the naturally occurring heterogeneity of human cancers.

**Fig. 3:**
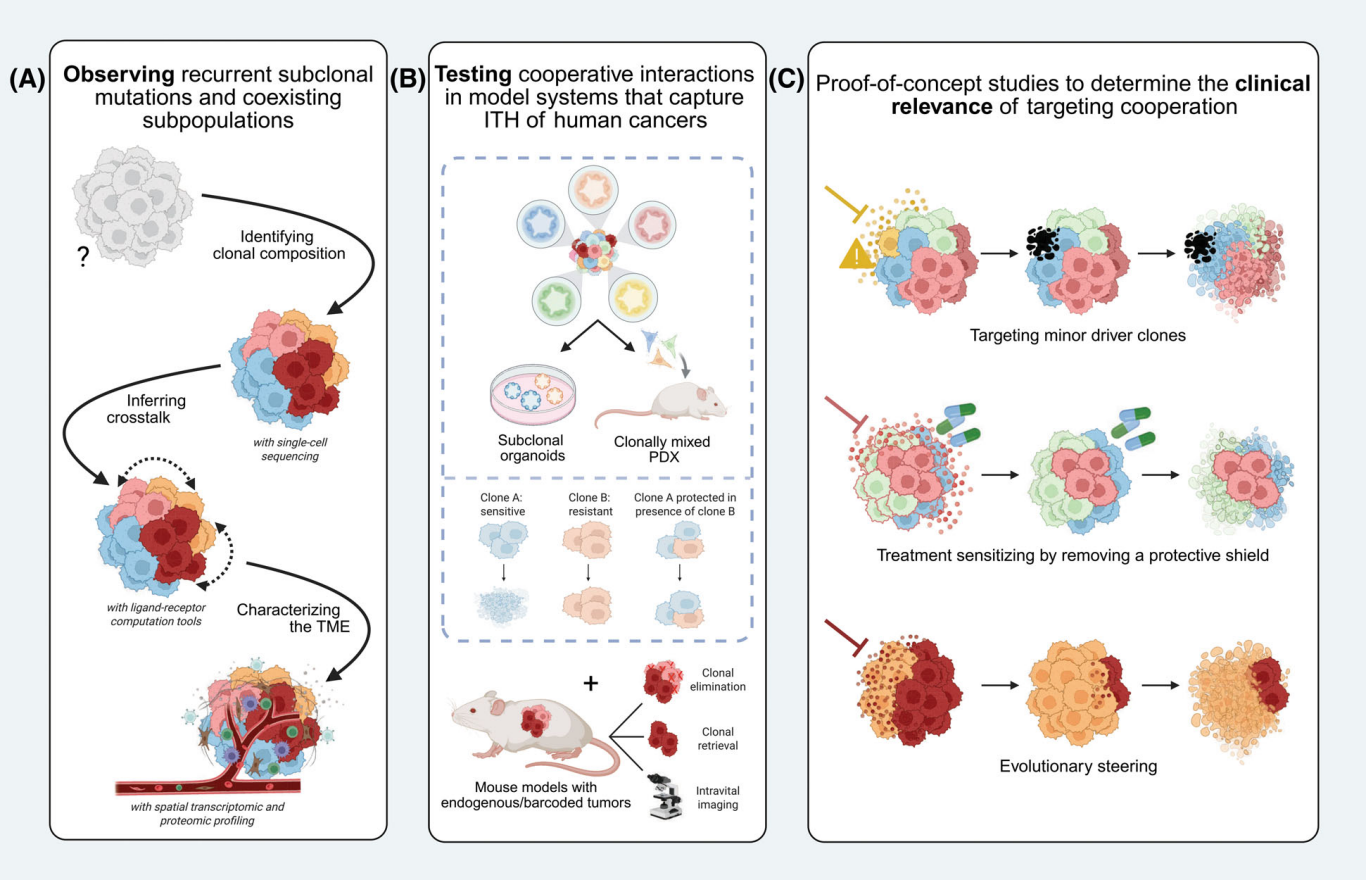
Suggested framework for future research. (A) Single‐cell sequencing studies, computational tools to infer crosstalk and spatial profiling (transcriptomics and proteomics) will identify and characterize recurrent subclonal mutations and coexisting subppopulations. TME, tumor microenvironment. (B) Harnessing the possibilities to dissect heterogeneity from human tumor samples and combine isolated clones in patient‐derived organoids and patient‐derived xenograft (PDX) models will allow formally proving cooperation. Mouse models carrying endogenous and/or barcoded tumors provides an additional approach that incorporates the genetic diversity and native microenvironment of the tumor. (C) Studies that assess the potential of different strategies to target clonal cooperation are needed to bridge the gap between observing clonal cooperativity and determining its clinical relevance: targeting minor driver clones to induce collateral damage, combining therapies to remove non‐cell‐autonomous protection from therapy and thereby sensitize tumor subpopulations, and steering the outgrowth of a tumor subpopulations that cannot survive in the absence of a previously coexisting subpopulation. Figure creating using BioRender.com.

We suggest a framework for future research based on three pillars. First, sequencing studies that characterize genetic and nongenetic ITH will enable the identification of recurrent subclonal alterations [1, 27], or phenotypic cell states that coexist within individual tumors [70]. This may be combined with computational tools to infer cellular crosstalk [106, 115], and leverage advances in spatial profiling to further define the relation of specific subclones to each other and their local microenvironment [33]. Of specific interest will be mutations that—based on their expected fitness advantage—are unexpectedly subclonal [9, 101]. Those may also become manifest after shifts in selective pressure [102], calling for sampling throughout the course of treatment (Fig. 3A) [127, 138].

Second, formally proving that two populations cooperate requires functional evidence. It will be crucial to develop models that can capture the heterogeneity inherent to human cancers. Organoids and PDX models better represent the heterogeneity of human cancers than traditional cell lines that have been in culture for decades. Importantly, both organoids and PDX have been generated from separate tumor regions and subclones [75, 139], providing a route toward separating and recombining individual subclones. This can form a unique study platform to assess their potential to collaborate in response to perturbations of interest. In addition to these reductionist approaches, there will be a need for mouse models carrying endogenous tumors with increased genetic diversity [140], to study cooperativity in its native microenvironment (Fig. 3B).

Ultimately, the promise of unveiling clonal cooperation is improving treatment outcome by targeting specific subpopulations or interactions. The success of this approach will be largely determined by the ability to identify the mediators of cooperativity at the molecular level, and the current evidence suggests that secreted factors or their receptors may be a particularly relevant class of molecules to target. There is a need for studies that assess the potential of targeting minor driver clones to induce collateral damage, combination therapies aimed at sensitizing populations by removing a protective shield cast by a (minor) subclone, the identification of subclonal targets, and evolutionary steering to drive essential subclones to extinction (Fig. 3C) [75]. Clearly, the road toward incorporating clonal interactions in clinical strategies is complex and this will not be realized overnight. However, we anticipate that a better understanding of clonal cooperativity may open the door to radically new treatment paradigms that destabilize the tumor and improve treatment outcome by finding and targeting its weakest link.

## Conflict of interest

EEV is founder and current member of the supervisory board of the Hartwig Medical Foundation, independent nonexecutive director of Sanofi, co‐founder of Mosaic Therapeutics, and board member and founder of the Center for Personalized Cancer Treatment.

## Author contributions

MCK contributed to the conceptualization and writing of the manuscript. MW contributed to the conceptualization, review and editing of the manuscript. EEV and KKD contributed to the conceptualization, review and editing of the manuscript, funding acquisition, and supervision.

## References

[1] Dentro SC , Leshchiner I , Haase K , Tarabichi M , Wintersinger J , Deshwar AG , et al. Characterizing genetic intra‐tumor heterogeneity across 2,658 human cancer genomes. Cell. 2021;184:2239–2254.e39. 10.1016/j.cell.2021.03.009 33831375 PMC8054914

[2] McGranahan N , Swanton C . Clonal heterogeneity and tumor evolution: past, present, and the future. Cell. 2017;168:613–628. 10.1016/j.cell.2017.01.018 28187284

[3] Caswell DR , Swanton C . The role of tumour heterogeneity and clonal cooperativity in metastasis, immune evasion and clinical outcome. BMC Med. 2017;15:133. 10.1186/s12916-017-0900-y 28716075 PMC5514532

[4] Zhou H , Neelakantan D , Ford HL . Clonal cooperativity in heterogenous cancers. Semin Cell Dev Biol. 2017;64:79–89. 10.1016/j.semcdb.2016.08.028 27582427 PMC5330947

[5] Bose D , Zimmerman LJ , Pierobon M , Petricoin E , Tozzi F , Parikh A , et al. Chemoresistant colorectal cancer cells and cancer stem cells mediate growth and survival of bystander cells. Br J Cancer. 2011;105:1759–1767. 10.1038/bjc.2011.449 22045189 PMC3242606

[6] Carneiro CS , Hapeman JD , Nedelcu AM . Synergistic inter‐clonal cooperation involving crosstalk, co‐option and co‐dependency can enhance the invasiveness of genetically distant cancer clones. BMC Ecol Evol. 2023;23:20. 10.1186/s12862-023-02129-7 37226092 PMC10207807

[7] Cleary AS , Leonard TL , Gestl SA , Gunther EJ . Tumour cell heterogeneity maintained by cooperating subclones in Wnt‐driven mammary cancers. Nature. 2014;508:113–117. 10.1038/nature13187 24695311 PMC4050741

[8] Hobor S , Van Emburgh BO , Crowley E , Misale S , Di Nicolantonio F , Bardelli A . TGFalpha and amphiregulin paracrine network promotes resistance to EGFR blockade in colorectal cancer cells. Clin Cancer Res. 2014;20:6429–6438. 10.1158/1078-0432.CCR-14-0774 24916700

[9] Inda MM , Bonavia R , Mukasa A , Narita Y , Sah DW , Vandenberg S , et al. Tumor heterogeneity is an active process maintained by a mutant EGFR‐induced cytokine circuit in glioblastoma. Genes Dev. 2010;24:1731–1745. 10.1101/gad.1890510 20713517 PMC2922502

[10] Janiszewska M , Tabassum DP , Castano Z , Cristea S , Yamamoto KN , Kingston NL , et al. Subclonal cooperation drives metastasis by modulating local and systemic immune microenvironments. Nat Cell Biol. 2019;21:879–888. 10.1038/s41556-019-0346-x 31263265 PMC6609451

[11] Marusyk A , Tabassum DP , Altrock PM , Almendro V , Michor F , Polyak K . Non‐cell‐autonomous driving of tumour growth supports sub‐clonal heterogeneity. Nature. 2014;514:54–58. 10.1038/nature13556 25079331 PMC4184961

[12] Wu M , Pastor‐Pareja JC , Xu T . Interaction between Ras (V12) and scribbled clones induces tumour growth and invasion. Nature. 2010;463:545–548. 10.1038/nature08702 20072127 PMC2835536

[13] de Visser KE , Joyce JA . The evolving tumor microenvironment: from cancer initiation to metastatic outgrowth. Cancer Cell. 2023;41:374–403. 10.1016/j.ccell.2023.02.016 36917948

[14] Tabassum DP , Polyak K . Tumorigenesis: it takes a village. Nat Rev Cancer. 2015;15:473–483. 10.1038/nrc3971 26156638

[15] Parker TM , Gupta K , Palma AM , Yekelchyk M , Fisher PB , Grossman SR , et al. Cell competition in intratumoral and tumor microenvironment interactions. EMBO J. 2021;40:e107271. 10.15252/embj.2020107271 34368984 PMC8408594

[16] Pelham CJ , Nagane M , Madan E . Cell competition in tumor evolution and heterogeneity: merging past and present. Semin Cancer Biol. 2020;63:11–18. 10.1016/j.semcancer.2019.07.008 31323289

[17] Ohsawa S , Sato Y , Enomoto M , Nakamura M , Betsumiya A , Igaki T . Mitochondrial defect drives non‐autonomous tumour progression through hippo signalling in Drosophila. Nature. 2012;490:547–551. 10.1038/nature11452 23023132

[18] Calbo J , van Moniort E , Proost N , van Drunen E , Beverloo HB , Meuwissen R , et al. A functional role for tumor cell heterogeneity in a mouse model of small cell lung cancer. Cancer Cell. 2011;19:244–256. 10.1016/j.ccr.2010.12.021 21316603

[19] Hershey BJ , Barozzi S , Orsenigo F , Pompei S , Iannelli F , Kamrad S , et al. Clonal cooperation through soluble metabolite exchange facilitates metastatic outgrowth by modulating Allee effect. Sci Adv. 2023;9:eadh4184. 10.1126/sciadv.adh4184 37713487 PMC10881076

[20] Kallberg J , Harrison A , March V , Berzina S , Nemazanyy I , Kepp O , et al. Intratumor heterogeneity and cell secretome promote chemotherapy resistance and progression of colorectal cancer. Cell Death Dis. 2023;14:306. 10.1038/s41419-023-05806-z 37142595 PMC10160076

[21] Kwon MC , Proost N , Song JY , Sutherland KD , Zevenhoven J , Berns A . Paracrine signaling between tumor subclones of mouse SCLC: a critical role of ETS transcription factor Pea3 in facilitating metastasis. Genes Dev. 2015;29:1587–1592. 10.1101/gad.262998.115 26215568 PMC4536306

[22] Le MT , Hamar P , Guo C , Basar E , Perdigao‐Henriques R , Balaj L , et al. miR‐200‐containing extracellular vesicles promote breast cancer cell metastasis. J Clin Invest. 2014;124:5109–5128. 10.1172/JCI75695 25401471 PMC4348969

[23] Naffar‐Abu Amara S , Kuiken HJ , Selfors LM , Butler T , Leung ML , Leung CT , et al. Transient commensal clonal interactions can drive tumor metastasis. Nat Commun. 2020;11:5799. 10.1038/s41467-020-19584-1 33199705 PMC7669858

[24] Obenauf AC , Zou Y , Ji AL , Vanharanta S , Shu W , Shi H , et al. Therapy‐induced tumour secretomes promote resistance and tumour progression. Nature. 2015;520:368–372. 10.1038/nature14336 25807485 PMC4507807

[25] Qin N , Paisana E , Langini M , Picard D , Malzkorn B , Custodia C , et al. Intratumoral heterogeneity of MYC drives medulloblastoma metastasis and angiogenesis. Neuro Oncol. 2022;24:1509–1523. 10.1093/neuonc/noac068 35307743 PMC9435486

[26] Yesmin F , Furukawa K , Kambe M , Ohmi Y , Bhuiyan RH , Hasnat MA , et al. Extracellular vesicles released from ganglioside GD2‐expressing melanoma cells enhance the malignant properties of GD2‐negative melanomas. Sci Rep. 2023;13:4987. 10.1038/s41598-023-31216-4 36973292 PMC10042834

[27] Frankell AM , Dietzen M , Al Bakir M , Lim EL , Karasaki T , Ward S , et al. The evolution of lung cancer and impact of subclonal selection in TRACERx. Nature. 2023;616:525–533. 10.1038/s41586-023-05783-5 37046096 PMC10115649

[28] Jamal‐Hanjani M , Wilson GA , McGranahan N , Birkbak NJ , Watkins TBK , Veeriah S , et al. Tracking the evolution of non‐small‐cell lung cancer. N Engl J Med. 2017;376:2109–2121. 10.1056/NEJMoa1616288 28445112

[29] Sottoriva A , Kang H , Ma Z , Graham TA , Salomon MP , Zhao J , et al. A big bang model of human colorectal tumor growth. Nat Genet. 2015;47:209–216. 10.1038/ng.3214 25665006 PMC4575589

[30] Suzuki Y , Ng SB , Chua C , Leow WQ , Chng J , Liu SY , et al. Multiregion ultra‐deep sequencing reveals early intermixing and variable levels of intratumoral heterogeneity in colorectal cancer. Mol Oncol. 2017;11:124–139. 10.1002/1878-0261.12012 28145097 PMC5527459

[31] Okonechnikov K , Joshi P , Korber V , Rademacher A , Bortolomeazzi M , Mallm JP , et al. Oncogene aberrations drive medulloblastoma progression, not initiation. Nature. 2025;642:1062–1072. 10.1038/s41586-025-08973-5 40335697 PMC12222029

[32] Erickson A , He M , Berglund E , Marklund M , Mirzazadeh R , Schultz N , et al. Spatially resolved clonal copy number alterations in benign and malignant tissue. Nature. 2022;608:360–367. 10.1038/s41586-022-05023-2 35948708 PMC9365699

[33] Lomakin A , Svedlund J , Strell C , Gataric M , Shmatko A , Rukhovich G , et al. Spatial genomics maps the structure, nature and evolution of cancer clones. Nature. 2022;611:594–602. 10.1038/s41586-022-05425-2 36352222 PMC9668746

[34] Taipale J , Keski‐Oja J . Growth factors in the extracellular matrix. FASEB J. 1997;11:51–59. 10.1096/fasebj.11.1.9034166 9034166

[35] Traister A , Shi W , Filmus J . Mammalian notum induces the release of glypicans and other GPI‐anchored proteins from the cell surface. Biochem J. 2008;410:503–511. 10.1042/BJ20070511 17967162

[36] Routledge D , Scholpp S . Mechanisms of intercellular Wnt transport. Development. 2019;146:dev176073. 10.1242/dev.176073 31092504

[37] Hoekstra ME , Bornes L , Dijkgraaf FE , Philips D , Pardieck IN , Toebes M , et al. Long‐distance modulation of bystander tumor cells by CD8(+) T cell‐secreted IFNgamma. Nat Cancer. 2020;1:291–301. 10.1038/s43018-020-0036-4 32566933 PMC7305033

[38] Doglioni G , Parik S , Fendt SM . Interactions in the (pre)metastatic niche support metastasis formation. Front Oncol. 2019;9:219. 10.3389/fonc.2019.00219 31069166 PMC6491570

[39] Mollaoglu G , Tepper A , Falcomata C , Potak HT , Pia L , Amabile A , et al. Ovarian cancer‐derived IL‐4 promotes immunotherapy resistance. Cell. 2024;187:7492–7510.e22. 10.1016/j.cell.2024.10.006 39481380 PMC11682930

[40] Ghazalpour A , Bennett B , Petyuk VA , Orozco L , Hagopian R , Mungrue IN , et al. Comparative analysis of proteome and transcriptome variation in mouse. PLoS Genet. 2011;7:e1001393. 10.1371/journal.pgen.1001393 21695224 PMC3111477

[41] Savage SR , Yi X , Lei JT , Wen B , Zhao H , Liao Y , et al. Pan‐cancer proteogenomics expands the landscape of therapeutic targets. Cell. 2024;187:4389–4407.e15. 10.1016/j.cell.2024.05.039 38917788 PMC12010439

[42] Zhang Y , Chen F , Chandrashekar DS , Varambally S , Creighton CJ . Proteogenomic characterization of 2002 human cancers reveals pan‐cancer molecular subtypes and associated pathways. Nat Commun. 2022;13:2669. 10.1038/s41467-022-30342-3 35562349 PMC9106650

[43] Eichelbaum K , Winter M , Berriel Diaz M , Herzig S , Krijgsveld J . Selective enrichment of newly synthesized proteins for quantitative secretome analysis. Nat Biotechnol. 2012;30:984–990. 10.1038/nbt.2356 23000932

[44] Ignacio BJ , Dijkstra J , Mora N , Slot EFJ , van Weijsten MJ , Storkebaum E , et al. THRONCAT: metabolic labeling of newly synthesized proteins using a bioorthogonal threonine analog. Nat Commun. 2023;14:3367. 10.1038/s41467-023-39063-7 37291115 PMC10250548

[45] Bennett HM , Stephenson W , Rose CM , Darmanis S . Single‐cell proteomics enabled by next‐generation sequencing or mass spectrometry. Nat Methods. 2023;20:363–374. 10.1038/s41592-023-01791-5 36864196

[46] Hartmann FSF , Gregoire M , Renzi F , Delvigne F . Single cell technologies for monitoring protein secretion heterogeneity. Trends Biotechnol. 2024;42:1144–1160. 10.1016/j.tibtech.2024.02.011 38480024

[47] Langerman J , Baghdasarian S , Cheng RY , James RG , Plath K , Di Carlo D . Linking single‐cell transcriptomes with secretion using SEC‐seq. Nat Protoc. 2025;20:2034–2055. 10.1038/s41596-024-01112-w 39979460

[48] Yuan S , Zhang P , Zhang F , Yan S , Dong R , Wu C , et al. Profiling signaling mediators for cell‐cell interactions and communications with microfluidics‐based single‐cell analysis tools. iScience. 2025;28:111663. 10.1016/j.isci.2024.111663 39868039 PMC11763584

[49] Guzelsoy G , Elorza SD , Ros M , Schachtner LT , Hayashi M , Hobson‐Gutierrez S , et al. Cooperative nutrient scavenging is an evolutionary advantage in cancer. Nature. 2025;640:534–542. 10.1038/s41586-025-08588-w 39972131 PMC11981941

[50] Peinado P , Stazi M , Ballabio C , Margineanu MB , Li Z , Colon CI , et al. Intrinsic electrical activity drives small‐cell lung cancer progression. Nature. 2025;639:765–775. 10.1038/s41586-024-08575-7 39939778 PMC11922742

[51] Davis JB , Krishna SS , Abi Jomaa R , Duong CT , Espina V , Liotta LA , et al. A new model isolates glioblastoma clonal interactions and reveals unexpected modes for regulating motility, proliferation, and drug resistance. Sci Rep. 2019;9:17380. 10.1038/s41598-019-53850-7 31758030 PMC6874607

[52] Jiang D , He J , Yu L . The migrasome, an organelle for cell‐cell communication. Trends Cell Biol. 2025;35:205–216. 10.1016/j.tcb.2024.05.003 38866683

[53] Osswald M , Jung E , Sahm F , Solecki G , Venkataramani V , Blaes J , et al. Brain tumour cells interconnect to a functional and resistant network. Nature. 2015;528:93–98. 10.1038/nature16071 26536111

[54] Saito K , Zhang Q , Yang H , Yamatani K , Ai T , Ruvolo V , et al. Exogenous mitochondrial transfer and endogenous mitochondrial fission facilitate AML resistance to OxPhos inhibition. Blood Adv. 2021;5:4233–4255. 10.1182/bloodadvances.2020003661 34507353 PMC8945617

[55] Williams JB , Li S , Higgs EF , Cabanov A , Wang X , Huang H , et al. Tumor heterogeneity and clonal cooperation influence the immune selection of IFN‐gamma‐signaling mutant cancer cells. Nat Commun. 2020;11:602. 10.1038/s41467-020-14290-4 32001684 PMC6992737

[56] Zhu M , Zou Q , Huang R , Li Y , Xing X , Fang J , et al. Lateral transfer of mRNA and protein by migrasomes modifies the recipient cells. Cell Res. 2021;31:237–240. 10.1038/s41422-020-00415-3 32994478 PMC8026638

[57] Borcherding N , Brestoff JR . The power and potential of mitochondria transfer. Nature. 2023;623:283–291. 10.1038/s41586-023-06537-z 37938702 PMC11590279

[58] Lim JS , Ibaseta A , Fischer MM , Cancilla B , O'Young G , Cristea S , et al. Intratumoural heterogeneity generated by notch signalling promotes small‐cell lung cancer. Nature. 2017;545:360–364. 10.1038/nature22323 28489825 PMC5776014

[59] Francis JM , Zhang CZ , Maire CL , Jung J , Manzo VE , Adalsteinsson VA , et al. EGFR variant heterogeneity in glioblastoma resolved through single‐nucleus sequencing. Cancer Discov. 2014;4:956–971. 10.1158/2159-8290.CD-13-0879 24893890 PMC4125473

[60] Vinci M , Burford A , Molinari V , Kessler K , Popov S , Clarke M , et al. Functional diversity and cooperativity between subclonal populations of pediatric glioblastoma and diffuse intrinsic pontine glioma cells. Nat Med. 2018;24:1204–1215. 10.1038/s41591-018-0086-7 29967352 PMC6086334

[61] Noble R , Burri D , Le Sueur C , Lemant J , Viossat Y , Kather JN , et al. Spatial structure governs the mode of tumour evolution. Nat Ecol Evol. 2022;6:207–217. 10.1038/s41559-021-01615-9 34949822 PMC8825284

[62] Archetti M , Ferraro DA , Christofori G . Heterogeneity for IGF‐II production maintained by public goods dynamics in neuroendocrine pancreatic cancer. Proc Natl Acad Sci USA. 2015;112:1833–1838. 10.1073/pnas.1414653112 25624490 PMC4330744

[63] Morrow RJ , Allam AH , Yeo B , Deb S , Murone C , Lim E , et al. Paracrine IL‐6 signaling confers proliferation between heterogeneous inflammatory breast cancer sub‐clones. Cancers (Basel). 2022;14:2292. 10.3390/cancers14092292 35565421 PMC9105876

[64] Laurent‐Puig P , Pekin D , Normand C , Kotsopoulos SK , Nizard P , Perez‐Toralla K , et al. Clinical relevance of KRAS‐mutated subclones detected with picodroplet digital PCR in advanced colorectal cancer treated with anti‐EGFR therapy. Clin Cancer Res. 2015;21:1087–1097. 10.1158/1078-0432.CCR-14-0983 25248381

[65] Househam J , Heide T , Cresswell GD , Spiteri I , Kimberley C , Zapata L , et al. Phenotypic plasticity and genetic control in colorectal cancer evolution. Nature. 2022;611:744–753. 10.1038/s41586-022-05311-x 36289336 PMC9684078

[66] Sharma A , Merritt E , Hu X , Cruz A , Jiang C , Sarkodie H , et al. Non‐genetic intra‐tumor heterogeneity is a major predictor of phenotypic heterogeneity and ongoing evolutionary dynamics in lung tumors. Cell Rep. 2019;29:2164–2174.e5. 10.1016/j.celrep.2019.10.045 31747591 PMC6952742

[67] Greenwald AC , Darnell NG , Hoefflin R , Simkin D , Mount CW , Gonzalez Castro LN , et al. Integrative spatial analysis reveals a multi‐layered organization of glioblastoma. Cell. 2024;187:2485–2501.e26. 10.1016/j.cell.2024.03.029 38653236 PMC11088502

[68] Marusyk A , Janiszewska M , Polyak K . Intratumor heterogeneity: the Rosetta stone of therapy resistance. Cancer Cell. 2020;37:471–484. 10.1016/j.ccell.2020.03.007 32289271 PMC7181408

[69] Torborg SR , Grbovic‐Huezo O , Singhal A , Holm M , Wu K , Han X , et al. Solid tumor growth depends on an intricate equilibrium of malignant cell states. *bioRxiv*. 2023. 10.1101/2023.12.30.573100 PMC13179057 41875882

[70] Barkley D , Moncada R , Pour M , Liberman DA , Dryg I , Werba G , et al. Cancer cell states recur across tumor types and form specific interactions with the tumor microenvironment. Nat Genet. 2022;54:1192–1201. 10.1038/s41588-022-01141-9 35931863 PMC9886402

[71] Pelka K , Hofree M , Chen JH , Sarkizova S , Pirl JD , Jorgji V , et al. Spatially organized multicellular immune hubs in human colorectal cancer. Cell. 2021;184:4734–4752.e20. 10.1016/j.cell.2021.08.003 34450029 PMC8772395

[72] Kok SY , Oshima H , Takahashi K , Nakayama M , Murakami K , Ueda HR , et al. Malignant subclone drives metastasis of genetically and phenotypically heterogenous cell clusters through fibrotic niche generation. Nat Commun. 2021;12:863. 10.1038/s41467-021-21160-0 33558489 PMC7870854

[73] Dijkstra KK , Cattaneo CM , Weeber F , Chalabi M , van de Haar J , Fanchi LF , et al. Generation of tumor‐reactive T cells by co‐culture of peripheral blood lymphocytes and tumor organoids. Cell. 2018;174:1586–1598.e12. 10.1016/j.cell.2018.07.009 30100188 PMC6558289

[74] Ramos Zapatero M , Tong A , Opzoomer JW , O'Sullivan R , Cardoso Rodriguez F , Sufi J , et al. Trellis tree‐based analysis reveals stromal regulation of patient‐derived organoid drug responses. Cell. 2023;186:5606–5619.e24. 10.1016/j.cell.2023.11.005 38065081

[75] Roerink SF , Sasaki N , Lee‐Six H , Young MD , Alexandrov LB , Behjati S , et al. Intra‐tumour diversification in colorectal cancer at the single‐cell level. Nature. 2018;556:457–462. 10.1038/s41586-018-0024-3 29643510

[76] Dijkstra KK , Vendramin R , Karagianni D , Witsen M , Galvez‐Cancino F , Hill MS , et al. Subclonal immune evasion in non‐small cell lung cancer. Cancer Cell. 2025;43:1833–1849.e10. 10.1016/j.ccell.2025.06.012 40614739

[77] Wolf Y , Bartok O , Patkar S , Eli GB , Cohen S , Litchfield K , et al. UVB‐induced tumor heterogeneity diminishes immune response in melanoma. Cell. 2019;179:219–235.e21. 10.1016/j.cell.2019.08.032 31522890 PMC6863386

[78] Martinez‐Jimenez F , Movasati A , Brunner SR , Nguyen L , Priestley P , Cuppen E , et al. Pan‐cancer whole‐genome comparison of primary and metastatic solid tumours. Nature. 2023;618:333–341. 10.1038/s41586-023-06054-z 37165194 PMC10247378

[79] Saito T , Niida A , Uchi R , Hirata H , Komatsu H , Sakimura S , et al. A temporal shift of the evolutionary principle shaping intratumor heterogeneity in colorectal cancer. Nat Commun. 2018;9:2884. 10.1038/s41467-018-05226-0 30038269 PMC6056524

[80] Gausachs M , Borras E , Chang K , Gonzalez S , Azuara D , Delgado Amador A , et al. Mutational heterogeneity in APC and KRAS arises at the crypt level and leads to polyclonality in early colorectal tumorigenesis. Clin Cancer Res. 2017;23:5936–5947. 10.1158/1078-0432.CCR-17-0821 28645942 PMC5626604

[81] Sadien ID , Adler S , Mehmed S , Bailey S , Sawle A , Couturier DL , et al. Polyclonality overcomes fitness barriers in Apc‐driven tumorigenesis. Nature. 2024;634:1196–1203. 10.1038/s41586-024-08053-0 39478206 PMC11525183

[82] Islam M , Yang Y , Simmons AJ , Shah VM , Musale KP , Xu Y , et al. Temporal recording of mammalian development and precancer. Nature. 2024;634:1187–1195. 10.1038/s41586-024-07954-4 39478207 PMC11525190

[83] Lu Z , Mo S , Xie D , Zhai X , Deng S , Zhou K , et al. Polyclonal‐to‐monoclonal transition in colorectal precancerous evolution. Nature. 2024;636:233–240. 10.1038/s41586-024-08133-1 39478225

[84] Lee‐Six H , Olafsson S , Ellis P , Osborne RJ , Sanders MA , Moore L , et al. The landscape of somatic mutation in normal colorectal epithelial cells. Nature. 2019;574:532–537. 10.1038/s41586-019-1672-7 31645730

[85] Martincorena I , Fowler JC , Wabik A , Lawson ARJ , Abascal F , Hall MWJ , et al. Somatic mutant clones colonize the human esophagus with age. Science. 2018;362:911–917. 10.1126/science.aau3879 30337457 PMC6298579

[86] Martincorena I , Roshan A , Gerstung M , Ellis P , Van Loo P , McLaren S , et al. Tumor evolution. High burden and pervasive positive selection of somatic mutations in normal human skin. Science. 2015;348:880–886. 10.1126/science.aaa6806 25999502 PMC4471149

[87] Yoshida K , Gowers KHC , Lee‐Six H , Chandrasekharan DP , Coorens T , Maughan EF , et al. Tobacco smoking and somatic mutations in human bronchial epithelium. Nature. 2020;578:266–272. 10.1038/s41586-020-1961-1 31996850 PMC7021511

[88] Demehri S , Turkoz A , Kopan R . Epidermal Notch1 loss promotes skin tumorigenesis by impacting the stromal microenvironment. Cancer Cell. 2009;16:55–66. 10.1016/j.ccr.2009.05.016 19573812 PMC2705757

[89] Sporn MB , Roberts AB . Autocrine growth factors and cancer. Nature. 1985;313:745–747. 10.1038/313745a0 3883191

[90] Joung JG , Oh BY , Hong HK , Al‐Khalidi H , Al‐Alem F , Lee HO , et al. Tumor heterogeneity predicts metastatic potential in colorectal cancer. Clin Cancer Res. 2017;23:7209–7216. 10.1158/1078-0432.CCR-17-0306 28939741

[91] Chapman A , Fernandez del Ama L , Ferguson J , Kamarashev J , Wellbrock C , Hurlstone A . Heterogeneous tumor subpopulations cooperate to drive invasion. Cell Rep. 2014;8:688–695. 10.1016/j.celrep.2014.06.045 25066122 PMC4542310

[92] Cheung KJ , Gabrielson E , Werb Z , Ewald AJ . Collective invasion in breast cancer requires a conserved basal epithelial program. Cell. 2013;155:1639–1651. 10.1016/j.cell.2013.11.029 24332913 PMC3941206

[93] Yoon SB , Chen L , Robinson IE , Khatib TO , Arthur RA , Claussen H , et al. Subpopulation commensalism promotes Rac1‐dependent invasion of single cells via laminin‐332. J Cell Biol. 2024;223:e202308080. 10.1083/jcb.202308080 38551497 PMC10982113

[94] Neelakantan D , Zhou H , Oliphant MUJ , Zhang X , Simon LM , Henke DM , et al. EMT cells increase breast cancer metastasis via paracrine GLI activation in neighbouring tumour cells. Nat Commun. 2017;8:15773. 10.1038/ncomms15773 28604738 PMC5472791

[95] Al Bakir M , Huebner A , Martinez‐Ruiz C , Grigoriadis K , Watkins TBK , Pich O , et al. The evolution of non‐small cell lung cancer metastases in TRACERx. Nature. 2023;616:534–542. 10.1038/s41586-023-05729-x 37046095 PMC10115651

[96] Birkbak NJ , McGranahan N . Cancer genome evolutionary trajectories in metastasis. Cancer Cell. 2020;37:8–19. 10.1016/j.ccell.2019.12.004 31935374

[97] Brady SW , McQuerry JA , Qiao Y , Piccolo SR , Shrestha G , Jenkins DF , et al. Combating subclonal evolution of resistant cancer phenotypes. Nat Commun. 2017;8:1231. 10.1038/s41467-017-01174-3 29093439 PMC5666005

[98] Findlay JM , Castro‐Giner F , Makino S , Rayner E , Kartsonaki C , Cross W , et al. Differential clonal evolution in oesophageal cancers in response to neo‐adjuvant chemotherapy. Nat Commun. 2016;7:11111. 10.1038/ncomms11111 27045317 PMC4822033

[99] Kreso A , O'Brien CA , van Galen P , Gan OI , Notta F , Brown AM , et al. Variable clonal repopulation dynamics influence chemotherapy response in colorectal cancer. Science. 2013;339:543–548. 10.1126/science.1227670 23239622 PMC9747244

[100] Janiszewska M , Liu L , Almendro V , Kuang Y , Paweletz C , Sakr RA , et al. In situ single‐cell analysis identifies heterogeneity for PIK3CA mutation and HER2 amplification in HER2‐positive breast cancer. Nat Genet. 2015;47:1212–1219. 10.1038/ng.3391 26301495 PMC4589505

[101] Misale S , Yaeger R , Hobor S , Scala E , Janakiraman M , Liska D , et al. Emergence of KRAS mutations and acquired resistance to anti‐EGFR therapy in colorectal cancer. Nature. 2012;486:532–536. 10.1038/nature11156 22722830 PMC3927413

[102] Salehi S , Kabeer F , Ceglia N , Andronescu M , Williams MJ , Campbell KR , et al. Clonal fitness inferred from time‐series modelling of single‐cell cancer genomes. Nature. 2021;595:585–590. 10.1038/s41586-021-03648-3 34163070 PMC8396073

[103] Karlsson K , Przybilla MJ , Kotler E , Khan A , Xu H , Karagyozova K , et al. Deterministic evolution and stringent selection during preneoplasia. Nature. 2023;618:383–393. 10.1038/s41586-023-06102-8 37258665 PMC10247377

[104] Archetti M . Collapse of intra‐tumor cooperation induced by engineered defector cells. Cancers (Basel). 2021;13:3674. 10.3390/cancers13153674 34359576 PMC8345189

[105] Pond KW , Doubrovinski K , Thorne CA . Wnt/beta‐catenin signaling in tissue self‐organization. Genes (Basel). 2020;11:939. 10.3390/genes11080939 32823838 PMC7464740

[106] Efremova M , Vento‐Tormo M , Teichmann SA , Vento‐Tormo R . CellPhoneDB: inferring cell‐cell communication from combined expression of multi‐subunit ligand‐receptor complexes. Nat Protoc. 2020;15:1484–1506. 10.1038/s41596-020-0292-x 32103204

[107] Jin S , Guerrero‐Juarez CF , Zhang L , Chang I , Ramos R , Kuan CH , et al. Inference and analysis of cell‐cell communication using CellChat. Nat Commun. 2021;12:1088. 10.1038/s41467-021-21246-9 33597522 PMC7889871

[108] Armingol E , Baghdassarian HM , Lewis NE . The diversification of methods for studying cell‐cell interactions and communication. Nat Rev Genet. 2024;25:381–400. 10.1038/s41576-023-00685-8 38238518 PMC11139546

[109] Jin S , Plikus MV , Nie Q . CellChat for systematic analysis of cell‐cell communication from single‐cell transcriptomics. Nat Protoc. 2025;20:180–219. 10.1038/s41596-024-01045-4 39289562

[110] Troule K , Petryszak R , Cakir B , Cranley J , Harasty A , Prete M , et al. CellPhoneDB v5: inferring cell‐cell communication from single‐cell multiomics data. Nat Protoc. 2025. 10.1038/s41596-024-01137-1 40133495

[111] Kumar MP , Du J , Lagoudas G , Jiao Y , Sawyer A , Drummond DC , et al. Analysis of single‐cell RNA‐Seq identifies cell‐cell communication associated with tumor characteristics. Cell Rep. 2018;25:1458–1468.e4. 10.1016/j.celrep.2018.10.047 30404002 PMC7009724

[112] Zhang M , Yang H , Wan L , Wang Z , Wang H , Ge C , et al. Single‐cell transcriptomic architecture and intercellular crosstalk of human intrahepatic cholangiocarcinoma. J Hepatol. 2020;73:1118–1130. 10.1016/j.jhep.2020.05.039 32505533

[113] Choi H , Sheng J , Gao D , Li F , Durrans A , Ryu S , et al. Transcriptome analysis of individual stromal cell populations identifies stroma‐tumor crosstalk in mouse lung cancer model. Cell Rep. 2015;10:1187–1201. 10.1016/j.celrep.2015.01.040 25704820

[114] Yeung TL , Sheng J , Leung CS , Li F , Kim J , Ho SY , et al. Systematic identification of druggable epithelial‐stromal crosstalk signaling networks in ovarian cancer. J Natl Cancer Inst. 2019;111:272–282. 10.1093/jnci/djy097 29860390 PMC6410941

[115] Li J , Hubisz MJ , Earlie EM , Duran MA , Hong C , Varela AA , et al. Non‐cell‐autonomous cancer progression from chromosomal instability. Nature. 2023;620:1080–1088. 10.1038/s41586-023-06464-z 37612508 PMC10468402

[116] Denisenko E , de Kock L , Tan A , Beasley AB , Beilin M , Jones ME , et al. Spatial transcriptomics reveals discrete tumour microenvironments and autocrine loops within ovarian cancer subclones. Nat Commun. 2024;15:2860. 10.1038/s41467-024-47271-y 38570491 PMC10991508

[117] Baldwin LA , Bartonicek N , Yang J , Wu SZ , Deng N , Roden DL , et al. DNA barcoding reveals ongoing immunoediting of clonal cancer populations during metastatic progression and immunotherapy response. Nat Commun. 2022;13:6539. 10.1038/s41467-022-34041-x 36344500 PMC9640547

[118] Guo Q , Spasic M , Maynard AG , Goreczny GJ , Bizuayehu A , Olive JF , et al. Clonal barcoding with qPCR detection enables live cell functional analyses for cancer research. Nat Commun. 2022;13:3837. 10.1038/s41467-022-31536-5 35788590 PMC9252988

[119] Umkehrer C , Holstein F , Formenti L , Jude J , Froussios K , Neumann T , et al. Isolating live cell clones from barcoded populations using CRISPRa‐inducible reporters. Nat Biotechnol. 2021;39:174–178. 10.1038/s41587-020-0614-0 32719478 PMC7616981

[120] Whiting FJH , Mossner M , Gabbutt C , Kimberley C , Barnes CP , Baker AM , et al. Quantitative measurement of phenotype dynamics during cancer drug resistance evolution using genetic barcoding. Nat Commun. 2025;16:5282. 10.1038/s41467-025-59479-7 40541962 PMC12181405

[121] Jang J , Ko KP , Zhang J , Jun S , Park JI . Deciphering precursor cell dynamics in esophageal preneoplasia via genetic barcoding and single‐cell transcriptomics. *bioRxiv*. 2025. 10.1101/2025.02.26.637920 PMC12704714 41337486

[122] Jin X , Demere Z , Nair K , Ali A , Ferraro GB , Natoli T , et al. A metastasis map of human cancer cell lines. Nature. 2020;588:331–336. 10.1038/s41586-020-2969-2 33299191 PMC8439149

[123] Oliveira EA , Milite S , Fernandez‐Mateos J , Cresswell GD , Yara‐Romero E , Vlachogiannis G , et al. Epigenetic heritability of cell plasticity drives cancer drug resistance through a one‐to‐many genotype‐to‐phenotype paradigm. Cancer Res. 2025;85:2921–2938. 10.1158/0008-5472.CAN-25-0999 40499006 PMC12314525

[124] Ishiguro S , Ishida K , Sakata RC , Ichiraku M , Takimoto R , Yogo R , et al. A multi‐kingdom genetic barcoding system for precise clone isolation. Nat Biotechnol. 2025. 10.1038/s41587-025-02649-1 PMC13090120 40399693

[125] Ma F , Guan Y , Yi Z , Chang L , Li Q , Chen S , et al. Assessing tumor heterogeneity using ctDNA to predict and monitor therapeutic response in metastatic breast cancer. Int J Cancer. 2020;146:1359–1368. 10.1002/ijc.32536 31241775

[126] Wang R , Yang Y , Ye WW , Xiang J , Chen S , Zou WB , et al. Case report: significant response to immune checkpoint inhibitor camrelizumab in a heavily pretreated advanced ER+/HER2‐ breast cancer patient with high tumor mutational burden. Front Oncol. 2020;10:588080. 10.3389/fonc.2020.588080 33634015 PMC7900143

[127] Williams MJ , Vazquez‐Garcia I , Tam G , Wu M , Varice N , Havasov E , et al. Tracking clonal evolution of drug resistance in ovarian cancer patients by exploiting structural variants in cfDNA. *bioRxiv*. 2024. 10.1101/2024.08.21.609031

[128] Koblan LW , Yost KE , Zheng P , Colgan WN , Jones MG , Yang D , et al. High‐resolution spatial mapping of cell state and lineage dynamics in vivo with PEtracer. Science. 2025;390:eadx3800. 10.1126/science.adx3800 40705858 PMC12766569

[129] Postrach D , Pritchard CEJ , Frank L , van Leeuwen T , Messal HA , Krimpenfort P , et al. Polytope: high‐resolution epitope barcoding for *in vivo* spatial fate‐mapping. *bioRxiv*. 2024. 10.1101/2024.11.20.624484

[130] Bollen Y , Stelloo E , van Leenen P , van den Bos M , Ponsioen B , Lu B , et al. Reconstructing single‐cell karyotype alterations in colorectal cancer identifies punctuated and gradual diversification patterns. Nat Genet. 2021;53:1187–1195. 10.1038/s41588-021-00891-2 34211178 PMC8346364

[131] Gao X , Huang X , Chen Z , Yang L , Zhou Y , Hou Z , et al. Supercontinuum‐tailoring multicolor imaging reveals spatiotemporal dynamics of heterogeneous tumor evolution. Nat Commun. 2024;15:9313. 10.1038/s41467-024-53697-1 39472437 PMC11522295

[132] Heinz MC , Peters NA , Oost KC , Lindeboom RGH , van Voorthuijsen L , Fumagalli A , et al. Liver colonization by colorectal cancer metastases requires YAP‐controlled plasticity at the micrometastatic stage. Cancer Res. 2022;82:1953–1968. 10.1158/0008-5472.CAN-21-0933 35570706 PMC9381095

[133] Tammela T , Sanchez‐Rivera FJ , Cetinbas NM , Wu K , Joshi NS , Helenius K , et al. A Wnt‐producing niche drives proliferative potential and progression in lung adenocarcinoma. Nature. 2017;545:355–359. 10.1038/nature22334 28489818 PMC5903678

[134] Al'Khafaji AM , Deatherage D , Brock A . Control of lineage‐specific gene expression by functionalized gRNA barcodes. ACS Synth Biol. 2018;7:2468–2474. 10.1021/acssynbio.8b00105 30169961 PMC6661167

[135] Feldman D , Tsai F , Garrity AJ , O'Rourke R , Brenan L , Ho P , et al. CloneSiser: enrichment of rare clones from heterogeneous cell populations. BMC Biol. 2020;18:177. 10.1186/s12915-020-00911-3 33234154 PMC7687773

[136] Gutierrez C , Al'Khafaji AM , Brenner E , Johnson KE , Gohil SH , Lin Z , et al. Multifunctional barcoding with ClonMapper enables high‐resolution study of clonal dynamics during tumor evolution and treatment. Nat Cancer. 2021;2:758–772. 10.1038/s43018-021-00222-8 34939038 PMC8691751

[137] Zhang ZY , Ding Y , Ezhilarasan R , Lhakhang T , Wang Q , Yang J , et al. Lineage‐coupled clonal capture identifies clonal evolution mechanisms and vulnerabilities of BRAF (V600E) inhibition resistance in melanoma. Cell Discov. 2022;8:102. 10.1038/s41421-022-00462-7 36202798 PMC9537441

[138] van de Haar J , Hoes LR , Roepman P , Lolkema MP , Verheul HMW , Gelderblom H , et al. Limited evolution of the actionable metastatic cancer genome under therapeutic pressure. Nat Med. 2021;27:1553–1563. 10.1038/s41591-021-01448-w 34373653

[139] Hynds RE , Huebner A , Pearce DR , Hill MS , Akarca AU , Moore DA , et al. Representation of genomic intratumor heterogeneity in multi‐region non‐small cell lung cancer patient‐derived xenogras models. Nat Commun. 2024;15:4653. 10.1038/s41467-024-47547-3 38821942 PMC11143323

[140] Hill W , Caswell DR , Swanton C . Capturing cancer evolution using genetically engineered mouse models (GEMMs). Trends Cell Biol. 2021;31:1007–1018. 10.1016/j.tcb.2021.07.003 34400045

